# Autism Spectrum Disorder: Genetic Mechanisms and Inheritance Patterns

**DOI:** 10.3390/genes16050478

**Published:** 2025-04-23

**Authors:** Ilaria La Monica, Maria Rosaria Di Iorio, Antonia Sica, Francesca Rufino, Chiara Sotira, Lucio Pastore, Barbara Lombardo

**Affiliations:** 1 Department of Molecular Medicine and Medical Biotechnologies, Federico II University, Via Sergio Pansini 5, 80131 Naples, Italy; lamonica@ceinge.unina.it (I.L.M.); sicaa@ceinge.unina.it (A.S.); rufino@ceinge.unina.it (F.R.); sotira@ceinge.unina.it (C.S.); lucio.pastore@unina.it (L.P.); 2 CEINGE-Biotecnologie Avanzate Franco Salvatore, Via G. Salvatore 486, 80145 Naples, Italy; diiorio@ceinge.unina.it

**Keywords:** autism spectrum disorder, copy number variant, genetic mechanisms, inheritance patterns

## Abstract

Autism spectrum disorder (ASD) is a neurodevelopmental disorder that first develops in early childhood and is characterized by restricted interests, activities, and behaviors, as well as difficulties with social interactions and communication. ASD arises from a complex interaction between environmental factors and genetic inheritance, influenced by epigenetic mechanisms. With an estimated heritability of 70–90%, ASD is highly familial, indicating that genetic factors play a significant role in its development. This shows how hundreds of genetic variants contribute to ASD, whose risk effects are highly variable and are often related to other conditions; these genetic alterations are at different levels, which include single gene mutations, monogenic disorders, genomic variants, and chromosomal abnormalities. Copy number variants (CNVs) appear to contribute significantly to understanding the pathogenesis of this complex disease. In some cases, single CNVs in genomic DNA are pathogenic and causative, supporting the hypothesis that some sporadic cases of ASD may result from rare mutations with significant clinical impact. However, in many cases, there are common genomic variants that increase the risk of developing ASD but are insufficient by themselves to determine an ASD phenotype, and rare genomic variants, of various sizes, inherited from a parent or de novo, that can be associated with the ASD phenotype. Therefore, the aim of this review is to deepen the concept of ASD inheritance through the two-hit theory of CNVs, in which the concomitant presence of two alterations could determine the clinical phenotypes, the concept of incomplete penetrance for inherited CNVs with pathogenic clinical significance, and the presence of compound heterozygosity. These aspects represent important mechanisms underlying the pathogenesis of autism, contributing to a better elucidation for the understanding of the genetic contribution to the ASD phenotype.

## 1. Introduction

**Figure 1:**
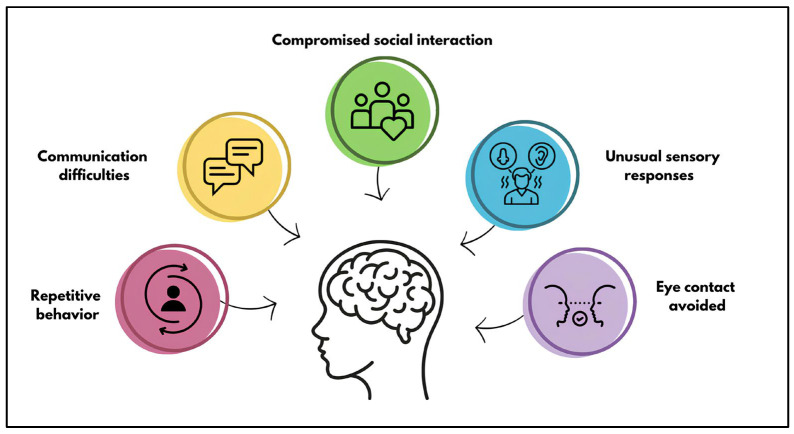
Main signs observed in patients affected by ASD.

Autism spectrum disorder (ASD) is a complex neurodevelopmental condition with onset during early development that is characterized by a range of conditions and symptoms, especially social and communication deficits, repetitive behaviors, and restricted interests; it varies from person to person not only in terms of symptoms and severity, but also in terms of the different subtypes of ASD (e.g., autistic disorder, Asperger’s syndrome, and pervasive developmental disorder not otherwise specified) [1,2,3,4,5,6]. The Diagnostic and Statistical Manual of Mental Disorders (DSM-5) (American Psychiatry, APA, Philadelphia, PA, USA, 2013), currently used for the diagnosis of autism, attributes the definition of “spectrum” to the condition of autism and uses criteria derived from diagnostic research assessment tools [7]. ASD has a prevalence of 1 in 160 children, with an increasing trend according to the World Health Organization (WHO) [8,9,10]. It is one of the most common groups of neurodevelopmental disorders (NDDs) and affects approximately 1–2% of the population [11,12,13]. ASD has an average male-female ratio of 4–5:1, showing an unequal distribution by gender [14,15,16]. Sex-differential biological factors and pathways that may account for the observed female protection and increased male risk in autism, predicted by the female protective effect model, are believed to involve the regulation and exposure to sex steroid hormones, as well as sex-differentiated activity in certain neural cell types [17]. According to Badcock et al., the key to understanding the variation in the sex ratio of ASD lies in the concept that both sexes possess maternal and paternal brains. ASD can arise either from the deterioration of the maternal brain (with a normal or enhanced paternal brain) or from the amplification of paternal brain effects (with a normal maternal brain). The deterioration of the maternal brain is expected to lead to more severe, “classic” ASD, often involving intellectual disability and language loss. In contrast, a normal maternal brain combined with enhanced paternal brain effects on cognition and behavior may result in “high-functioning” ASD or Asperger’s syndrome [18,19]. This agrees with Baron-Cohen’s extensive evidence on gender differences that describes males normally showing a bias towards stronger paternal brain effects, which predisposes them to ASD [19]. The intellectual disability and loss of language characteristic of classic ASD fit this prediction, but also necessarily generate a profound loss of phenotypic traits, making further evaluation in this context difficult, which is why further studies are underway [18,20]. In recent years, there has been growing interest in exploring the potential need for sex-differential diagnostic criteria for ASD [21]. As indicated in the DSM-5, individuals with ASD are characterized by persistent deficits in communication and social engagement, as well as restricted and repetitive patterns of behavior, interests, or activities. Specifically, deficits may be observed in areas such as social-emotional reciprocity, nonverbal communication behaviors used in social interactions, and the ability to establish, maintain, and understand age-appropriate relationships. According to the DSM-5, symptoms may be masked during early development and fully appear only when social demands exceed limited capacities or may be hidden by learned strategies in later life (Figure 1).

A clinically significant impairment in social, professional, or other critical areas of present functioning should result from the disability [7]. Furthermore, the DSM-5 explicitly recognizes that ASD may be accompanied by other disorders, including genetic disorders (i.e., fragile-X syndrome) and psychiatric conditions (i.e., attention-deficit/hyperactivity disorder [ADHD] or intellectual disability [ID]) [15]. Indeed, every individual with ASD shows some form of comorbidity, and at a subclinical level, twin studies suggest that autistic traits are linked to ADHD symptoms and that this association is due to a common genetic etiology [22]. ASD is considered a complex disorder with multifactorial etiology, in which genetic and non-genetic influences acting either alone or in combination contribute to the development of ASD. A complex interaction between environmental and genetic factors play an important role in the etiology of this condition, as is highlighted from many epidemiological studies [16,23]; in fact, it is currently hypothesized that the genetic-environment interaction also determines epigenetic alterations which could be partly responsible for the development of autism spectrum disorder in many patients [24].

ASD is highly familial, suggesting that genetic factors play a significant role in its development. In fact, it is considered one of the most heritable mental disorders, with studies reporting a heritability of more than 90% for autistic disorder [25,26]. This has been demonstrated through twin studies. Research by J. N. Constantino et al. found that the concordance rate of ASD in monozygotic (MZ) twins is between 70 and 90%, compared to 30 and 40% in dizygotic (DZ) twins, and 3 and 19% in siblings. Notably, the concordance rate is twice as high in siblings with both parents in common compared to those with only one parent in common [27]. The genetic heritability of a trait is often estimated by comparing the phenotypic concordance between MZ and DZ twins, who share 100% and 50% of their genetic material, respectively. A larger difference in concordance between MZ and DZ twins suggests a higher genetic contribution to the trait. The genetic basis of ASD has been further confirmed by the high concordance of clinical manifestations in MZ twins compared to DZ twins [28,29]. Therefore, it is widely accepted that genetic heritability plays a crucial role in the onset of ASD [1]. According to some studies, the prevalence of de novo chromosomal rearrangements is higher in autistic subjects from families with only one affected individual than in autistic subjects from families with more affected individuals [30,31]. Further studies have subsequently demonstrated that familial aggregation of subclinical autistic traits can occur only in families with more affected individuals, suggesting differential mechanisms of genetic transmission of ASD in the population [22,32,33,34]. Therefore, aggregation within families is better explained by shared genes rather than a shared environment. In this way, it is confirmed that the variation of autistic traits in the general population is highly heritable, even if the results are heterogeneous [22,34]; in fact, the risk of ASD increased with increasing genetic parentage [35]. A study by Bai et al. [36] has shown that a random underestimation of ASD can lead to an undervaluation of the true heritability and increase the contribution of the observed shared environment. Overall, twin studies provide strong evidence of a predominantly genetic contribution to ASD and negligible shared environmental effects, suggesting that ASD can be considered as a continuously distributed condition in the population with heritable traits, with similar but not identical genetic contributions [37,38]. Family studies indicate that the study of genomics can become a medical marker for ASD [38]; therefore, it is necessary to investigate the genetic factors underlying the disorder. Based on these considerations, in this review, we deepen the concept of ASD heritability for a better understanding of the genetic contribution underlying the phenotype. In particular, we emphasize the two-hit theory, which suggests that the simultaneous presence of two genetic alterations could influence the development of clinical phenotypes. Additionally, we discuss the concept of incomplete penetrance for inherited alterations with pathogenic clinical significance, as well as the role of compound heterozygosity in ASD.

## 2. ASD Genetics

Advances in genetic technology and testing used to identify possible causative factors in patients with ASD have led to the identification of a specific etiology in 40% of patients, consistently reporting chromosomal abnormalities in individuals with ASD. The advent of molecular cytogenetic testing modalities such as array comparative genomic hybridization (a-CGH) has improved the diagnostic power of genetic evaluations [39,40,41,42]. When compared to other genetic tests, the a-CGH analysis has shown the highest diagnostic yield in patients with ASD. To check for structural chromosomal patterns, such as deletions or duplications or heterozygosity patterns, in subjects with neurodevelopmental disorders, including ASD, high-resolution microarrays use both polymorphic DNA probes and CNV probes [43,44,45]. In fact, microarrays are first-line genetic tests and are most effective in detecting alterations that may be associated with the chromosome level [46]. In addition, next-generation sequencing (NGS) allows the identification of genetic changes that cannot be detected with microarray analysis. Therefore, single-nucleotide variants (SNVs) and copy number variants (CNVs) are among the genetic variants identified as the most likely to cause ASD; these variations can be inherited or de novo [47,48]. Common genetic variations are associated with small increases in risk and are frequently observed in the general population. However, current estimates suggest that these common variants account for only 12% of the variance in ASD risk [49]. Genetic models of ASD indicate a complex pattern of inheritance. While rare variants have a larger individual effect, they are not yet considered definitive causes for the onset of ASD. Similarly, common variants contribute only a limited amount to the overall risk of developing the disorder [15]. About 10% of people with ASD without a positive family history (known as simplex or sporadic autistic patients) show genetic defects, such as deletions or duplications, when studied at the chromosomal level, particularly using chromosomal microarray analysis. Instead, multiplex autistic individuals have a positive family history and, compared to typically developing children considered controls, they are more likely to exhibit multiple individual defects at the genetic level. About 20% of people with ASD have chromosomal deletions involving one or more genes, which account for the majority of CNVs. Hundreds of DNA polymorphisms have been identified in ASD risk gene loci across all chromosomes in the human genome, which contains approximately 20,000 genes. These findings are supported by both genome-wide association studies (GWAS) and genetic linkage analyses [46,50].

### Syndromic and Non-Syndromic ASD

ASD can be categorized into two types: syndromic, associated with other neurological disorders or syndromes, and non-syndromic. In fact, 5 to 10% of people with ASD have a specific monogenic genetic syndrome. Most of these conditions include a gene that controls the expression of other genes that are involved in the development of the central nervous system (CNS) in different ways [51,52,53,54,55,56]. Many children with ASD have some degree of learning disability, and genetic disorders associated with it have also been associated with ASD [57,58]. Originally, this dichotomy referred exclusively to the behavioral characteristics of the condition; their new definition indicates either the presence of comorbid phenotypic features or a genetic etiology associated with the onset of ASD. In the first instance, there is disagreement over which phenotypes (dysmorphic features, congenital malformations, or ID) define the comorbidity [59,60]. Regarding the genetic etiology of ASD, it is sometimes characterized as monogenic, while in other cases, it results from chromosomal and structural genomic abnormalities, such as CNVs. Some individuals with ASD may not exhibit obvious comorbidities, with their condition potentially linked to a single genetic cause. In contrast, many individuals with ASD, despite lacking a single identifiable genetic cause, show clear comorbidities [61,62,63]. Syndromic ASD is usually associated with conditions such as fragile X syndrome (FXS), tuberous sclerosis, or Rett syndrome, with phenotypes caused by a mutation in a particular gene or set of genes [64]. FXS is the most common cause of inherited learning disability; it is an X-linked dominant disorder with reduced penetrance caused by a dynamic mutation involving an unstable CGG trinucleotide expansion in the *FMR1* gene. Approximately 90% of male children with FXS exhibit one or more features of ASD (e.g., atypical social interaction, lack of eye contact, social anxiety and avoidance, perseverative speech, stereotyped behavior) [65,66]. Another common syndromic condition is tuberous sclerosis, which affects approximately 1% of individuals with ASD. It is an autosomal dominant inherited genetic disorder caused by mutations in one of the *TSC1* or *TSC2* tumor suppressor genes. Among the genes associated with ASD is *MECP2*, whose mutations are responsible for Rett syndrome, an X-linked dominant postnatal neurodevelopmental disorder. Rett syndrome is the second most common cause of severe cognitive impairment in females, after Down syndrome [67]. A structural genetic abnormality most commonly associated with syndromic ASD is the deletion in the 15q11q13 region. When this deletion occurs on the paternal chromosome, it causes Prader-Willi syndrome (PWS), whereas a deletion on the maternal chromosome results in Angelman syndrome. Recent studies of children with PWS have shown repetitive behaviors and social deficits that resemble ASD. However, it appears that the risk of autistic symptoms is higher when PWS results from maternal uniparental disomy, as opposed to the deletion of the paternal chromosome [68,69,70]. Another syndrome associated with CNVs is Williams syndrome, an autosomal dominant inherited neurodevelopmental disorder due to a 1.5 Mb microdeletion in the 7q11.23 region or 22q13.3 deletion syndrome characterized by typical ASD symptoms [71,72]. The conditions discussed above are therefore called syndromic forms of ASD or high-penetrance ASD. However, new genetic and biological discoveries have highlighted that there is no clear distinction between rare monogenic ASD and common multifactorial ASD. Since it is difficult to identify the disease genes for non-syndromic forms of ASD, due to the large number of genes and environmental factors that may contribute to its etiology, multiple causative loci have been identified. The 1q21.1, 15q13.3, 16p11.2, 17p11.2, 22q11.2, 16p13.1, and 7q11.23 (Table 1) regions represent the CNVs most linked to ASD [14,73,74].

The most common deletions, among clinically significant fully penetrant deletions, according to Moreno-De-Luca et al., include the 15q13.2–q13.3 region, followed by a small percentage of deletions at 3q29, 5q35 (Sotos syndrome region), and 22q11.2 (DiGeorge syndrome region) regions. The deletions of 1q21, 17q12, 16p12.1, 16p13.11, and 1q21 regions are included in the category of incomplete penetrance alterations and, among clinically significant fully penetrant duplications, the most frequent gain involves the 15q11.2–q13.3 region, a well-known genetic ASD anomaly, followed by gain of the 7q11.23 region, which has also been strongly associated with ASD. Duplications with incomplete complete penetrance involved 22q11.2, 1q21.1, and 15q13.2–q13.3 regions. Through a nested case-control analysis, the significant association between specific recurrent CNVs and ASD was also demonstrated [73]. The most common recurrent CNVs associated with ASD are microdeletions and microduplications in the 16p11.2 region, at frequencies of 0.42% and 0.39%, respectively, which are identified in approximately 1% of individuals with ASD [75,76,77]. Moreover, people with sporadic ASD are more likely to have rare de novo CNVs than autistic cases with a sibling affected. One of the first studies showing a link between de novo CNVs and ASD was carried out by Sebat et al., suggesting that rare de novo CNVs may be important risk factors for ASD, especially in patients who have sporadic disorder [78]. According to the study by Doelken et al., most of the CNVs contain several genes that could interact to produce the ASD phenotype [79] because they are functionally related to the development of the CNS, which may contribute to determining the phenotype.

## 3. ASD Inheritance Pattern

### 3.1. Double Hit Model

**Figure 2:**
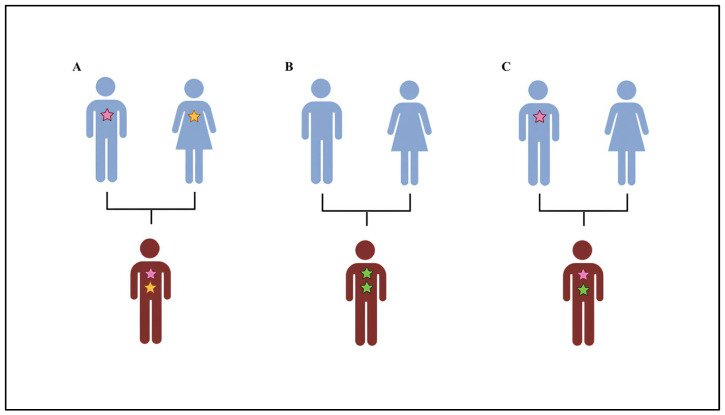
Double hit model. The risk of ASD can be caused by the presence of (**A**) two inherited alterations: one of paternal origin and one of maternal origin. (**B**) Two de novo alterations. (**C**) Two alterations: one de novo and one of parental origin.

The “double hit” hypothesis proposes that the presence of multiple CNVs within an individual’s genome can interact to produce clinical outcomes that are more severe or qualitatively different than those associated with a single CNV. This concept emphasizes the synergistic effects of genetic alterations in driving the phenotypic complexity observed in certain NDDs. Although most NDD-causative CNVs are de novo, growing evidence suggests that CNVs with high risk for NDDs show incomplete penetrance as they can be transmitted from unaffected parents to affected children (Figure 2).

According to Velinov et al., a possible explanation for the observed inconsistency of genotype-phenotype correlation may result from complex interactions between potentially pathogenic CNVs and additional/secondary CNVs or single-nucleotide variants, which together may contribute to disease development [80]. In line with this hypothesis, Girirajan et al. [81] and Duyzend et al. [82] observed that affected persons with a microdeletion on chromosome 16p12.1 are more likely to have additional large copy-number variants than healthy controls. Duyzend et al. identified secondary CNVs in individuals with 16p11.2 CNVs that affected genes associated with ASD risk and intellectual disability. For instance, deletions or duplications were observed in genes such as *CACNA2D3*, *TRIO*, and *KATNAL2*. Similarly, Pizzo et al. studied a large cohort of individuals with the 16p11.2 microdeletion and their families, using whole-exome sequencing and SNP analysis to identify rare sequence variants. They found that the presence of these variants correlated with disease severity and was more pronounced in families with a stronger history of the disorder. The authors suggested that these secondary variants are likely to affect relevant genes, modifying the pathogenicity of the CNV [83].

Secondary genetic events transform the 16p12.1 deletion phenotype from the normal development observed in carrier parents to the severe developmental phenotypes observed in their affected children; there are several hypotheses to explain this. As discussed by Veltman et al., one proposed explanation is that the two genomic alterations affect independent functional modules, and their combined effects cumulatively result in developmental delay (additive model of double hit). Alternatively, a distinct hypothesis suggests that, rather than a purely additive effect, the second event may contribute to the clinical phenotype by disrupting the same biological pathway, possibly amplifying its impact. This implies a model where both genetic events affect the same functional module (epistatic model of double hit). For example, converging evidence indicates that alterations in multiple genes encoding Rho GTPases can contribute to the development of intellectual disability. Similarly, loss-of-function monoallelic mutations in the sodium channels genes *CACNA1A* and *SCN8A* are commonly associated with various clinical features, including movement disorder, ID, ASD, and benign familial infantile seizures [84].

The concept of epistasis was first defined by Bateson as the masking or modifying influence of one allele on another located at a different locus. Later, Fisher refined the concept in quantitative terms, describing epistasis as a deviation from the additive effects of two genetic variants on a phenotypic trait [85,86]. According to Webber, epistasis occurs when the combination of alleles results in a phenotypic effect that deviates from the sum of their independent effects. Notably, epistasis can be either synergistic, where the combined effect is amplified, or antagonistic, where the combined effect is diminished. Epistatic interactions are closely linked to the concept of mutational load, which suggests that the penetrance and complexity of a disease phenotype are influenced by the number of disruptive genetic events [87]. In the same way, Girirajan et al. evaluated the generalizability of the double hit model by analyzing the genomic context of 72 large, rare CNVs either known to be associated with genomic disorders or potentially linked to disease. Their study revealed that among 2312 affected children carrying a primary variant, 200 individuals (8.7%; 87 girls and 113 boys) harbored at least one additional large variant affecting an autosome. Moreover, they observed a significant enrichment of second-site variants in children with phenotypically variable genomic disorders compared to those with syndromic genomic disorders. They further demonstrated that the presence of two large variants (exceeding 500 kb) of unknown significance was eight times more likely in children with developmental delay or intellectual disability compared to controls. This finding supported the applicability of the double hit model also for unknown significance CNVs. Investigating the inheritance patterns of CNVs, the authors identified a significant bias toward maternal inheritance of second-site variants. Additionally, a qualitative clinical reassessment confirmed that among children with the same genomic disorder, those carrying multiple variants exhibited deficits across more developmental domains compared to those with a single variant. Finally, the study showed that a greater number of affected genes correlated with lower IQ levels in a cohort of children with ASD [88].

According to Servetti et al., bioinformatic analyses revealed that genes involved in co-occurring CNVs often have synergistic roles in biological processes fundamental to neurodevelopment. For example, they describe a patient with a *CNTNAP2* deletion inherited from the mother and an *LRRC4C* deletion inherited from the father [89]. Both genes are involved in synapse formation, and their combined disruption may underlie the observed neurodevelopmental disorder [90]. The analysis of the “double hit” hypothesis has led the scientists to the identification of novel candidate genes for NDDs, including *PTPRD*, *BUD13*, *GLRA3*, *MIR4465*, *ABHD4*, and *WSCD2*. When these genes interact with other variants, they contribute significantly to the development of complex phenotypes. Furthermore, Velinov states that many studies have demonstrated the additive contribution of sequence variants, located outside the primary CNV, to the overall risk of ASD and cognitive disabilities [80]. The suggested mechanisms underlying these effects include the involvement of genes outside the CNV region, as well as the unmasking of recessive disorders affecting genes within the CNV [89,91,92]. In conclusion, the “double hit” hypothesis has profound implications for both diagnosis and genetic counseling. The interpretation of variants of uncertain significance (VUS) should consider their potential interactions with other genetic variants. The coexistence of multiple variants, even if individually non-pathogenic, may hold the key to understanding a patient’s phenotype [89].

Table 2 summarizes the main evidence supporting the double-hit model described above.

### 3.2. Modifier Genes

Genotype–phenotype correlation studies, as outlined by Guo et al., have revealed that individuals with mutations in multiple genes are at a significantly higher risk of developing neurodevelopmental disorders. Furthermore, a positive correlation exists between the cumulative number of disruptive genetic events and both the severity and the number of clinical manifestations observed. Additionally, the cumulative burden of common genetic variants may act as a primary predisposing factor, increasing the vulnerability of the genetic background to subsequent pathological events [93]. This suggests that common variants may function as modifiers that, in combination with other mutations, increase the risk of NDDs. As described by Bourgeron et al., NDDs are often associated with mutations in genes critical for brain development: neurexins (*NRXN*), neuroligins (*NLGN*), SHANKs, and cell-adhesion molecules (CAMs) have been implicated in NDDs through independent studies in both patients and mouse models [94]. As stated by Parenti et al., these genes exhibit a functional and bidirectional connection with numerous other genes associated with NDDs, highlighting the modifying role of potentially any genetic variant that affects other NDD-linked signaling cascade genes on the clinical outcome. Zaslavsky et al. conducted a recent transcriptomic analysis of human neurons with *SHANK2* mutations, revealing a significant presence of Fragile X RNA-binding protein (FMRP) targets and genes involved in chromatin and transcriptional regulation among the differentially expressed genes. Similarly, data from Huang et al. demonstrate a dysregulation of the PI3K pathway in neurons with reduced *SHANK3* expression. These findings suggest that mutations in *SHANK2* may impact additional molecular pathways [95,96,97].

Qin et al., conducted studies on mouse models of *SHANK3* haploinsufficiency, revealing abnormal levels of histone acetylation. Notably, when these alterations were corrected through histone deacetylase inhibition, the social deficits observed in the models were partially ameliorated. This finding underscores the interplay between synaptic scaffold proteins and epigenetic modifications, suggesting that epigenetic changes may modulate the impact of *SHANK3* mutations [98]. According to Ronan et al., many genes associated with neurodevelopmental disorders belong to the category of transcriptional regulators or chromatin remodelers. These proteins play a crucial role in regulating the maturation of both inhibitory and excitatory cortical connections during development [99]. Notable examples of genes linked to diseases and classified as chromatin remodelers or transcriptional regulators include *MECP2*, *SETD5*, *CHD8*, *ASH1L*, *ARID1B*, and *KMT2A.* Given the broad range of targets for each of these proteins, their dysregulation can result in pleiotropic effects [100,101]. From a therapeutic perspective, targeting these crucial networks may be able to ameliorate certain clinical aspects linked to NDDs, as suggested by the functional convergence of genetic variables underpinning these problems. Furthermore, by predicting (at least in vitro) how drugs would affect patient-specific molecular pathways, models made from human induced pluripotent stem cells hold potential for enabling tailored treatments.

Table 3 lists, by category, the genes previously described as modifier genes.

### 3.3. Incomplete Penetrance Model

The syndromic diseases are the ones in which the phenotypic features are numerous and the same molecular alteration associates with different conditions, such as epilepsy, schizophrenia (SCZ), ASD, ID, and congenital malformations. The type and the severity of the symptoms may be explained by additional rare genetic events and their inheritance in the families. The type and quantity of CNVs associated with external stimuli during development determine the different degrees of severity and outcome of the disease. Penetrance is defined as the percentage of individuals with phenotypic features having a disease-causing genotype, and incomplete penetrance describes how some carriers of CNVs do not develop any disorder [102,103].

It has been demonstrated that some ASD-associated CNVs are inherited from an unaffected parent or are found in control populations, exhibiting varying levels of penetrance. Although hundreds of loci have been linked to ASD, only 25–35% of cases have identified genetic variants as etiological factors, and incomplete penetrance further complicates the determination of ASD’s cause. Several studies have shown that the mechanism of incomplete penetrance contributes to the variability in the phenotype [14,94,104]. The study conducted by de Masfrand et al. presents 12 patients affected by NDDs, carrying a pathogenic or likely pathogenic loss-of-function variant in 11 genes, all known to cause neurodevelopmental phenotype with a high penetrance; all the variants are inherited from a parent evaluated as asymptomatic [102]. For some of the observed genes, there is no knowledge of incomplete penetrance, but for one of them, the *CAMTA1* gene, Jacobs et al. described one variant inherited from an apparently healthy mother in a cohort of 19 patients. Van Rahden et al. in a study detected in two patients a variant in *NDUFB11* gene inherited from an asymptomatic mother. Finally, Latchman et al. reported a family with a father and two kids, who shared the same variant of the *ZMIZ1* gene but exhibited significant phenotypic variability [105,106,107]. The molecular aspects of such mechanisms are still unclear, but several studies have pointed out important reflections, such as the need to integrate the concept of incomplete penetrance in the method for variant interpretation. The authors suggest classifying the SNVs and multi-nucleotide variants (MNVs) inherited from healthy parents as pathogenic if clinical and molecular arguments converge. Additionally, segregation studies should be systematically proposed and conducted to assess the molecular status of the parents, even if they are considered asymptomatic based on anamnestic evaluations. While carriers may show no symptoms, several studies have reported a correlation between CNVs and cognitive abilities. For example, Kendall et al. demonstrated that cognitive tests conducted on a group of asymptomatic individuals who were carriers of neurodevelopmental CNVs yielded intermediate results, compared to a group of non-carriers and a group of patients diagnosed with schizophrenia. Furthermore, they have a lower level of education and an employment status that does not require high professional training, compared to non-carriers [108]. The authors stated that there was a strong correlation between the penetrance of NDD CNVs and cognitive test results among unaffected adult carriers, suggesting that they may have difficulty forming families and relationships, most likely due to a confluence of issues, such as behavioral, medical, and cognitive. Other studies describe the event of incomplete penetrance by referring to CNVs related to ASD, in particular to the syndromic form of the latter. Among CNVs strongly associated with ASD, deletions in the 15q13.3 region clearly show incomplete penetrance as demonstrated by Furukawa et al. through a study on siblings, both affected and with the same microdeletion in the 15q13.3 region, which includes the *CHRNA7* gene, which encodes the α-7 subunit of the neuronal nicotinic acetylcholine receptor. The same deletion was also present in the healthy mother [103,109]. In their manuscript Mollon et al. discussed how whole and exome sequencing studies have shown that the clinical outcomes in some individuals with specific CNVs (1q21.1, 7q11.23, 16p12.1, 16p11.2, and 22q11.2) are influenced by additional rare variants in the genetic background, therefore, the presence of multiple variants may be an additional cause of the patient’s phenotype and incomplete penetrance interferes with the variability of the phenotype [103]. Goh et al. conducted a systematic review to determine the penetrance estimates for 92 CNVs associated with neurodevelopmental processes. Using the Bayesian formula, the authors compared data from the aggregated affected cohorts to the control cohort provided by gnomAD v4.0 CNV.

Through this analysis, the authors classified CNVs as pathogenic or non-pathogenic based on penetrance estimates and statistical significance: for example, the distal duplication of 22q11.2 shows a penetrance of 51% with a confidence interval of 32–77%, highlighting its pathogenic potential. Other CNVs identified as pathogenic include the duplication of 15q24, the deletion and duplication of 15q24.2q24.5, the duplication of 17q11.2, and the duplication of 17q21.31. In contrast, some CNVs were classified as benign, such as the duplication of 15q11.2 and the proximal duplication of 2q13. The authors also identified CNVs that showed no significant difference in frequency between affected individuals and controls. These findings suggest that these CNVs are unlikely to be pathogenic or may exhibit very low penetrance, as exemplified by the 2q11.2 deletion and proximal 2q13 duplications. Furthermore, the authors reported that certain CNVs typically considered to have full penetrance, such as the 7q11.23 deletion (Williams-Beuren Syndrome) and the 22q11.2 deletion (Velocardiofacial Syndrome), display a penetrance rate below 100% [110].

Table 4 summarizes the studies described above in support of the incomplete penetrance model for ASD.

### 3.4. Compound Heterozygosity

**Figure 3:**
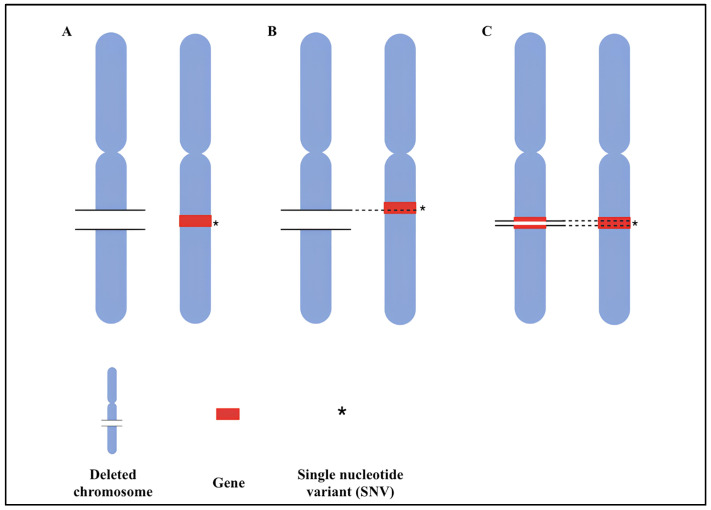
Different compound heterozygosity mechanisms. (**A**) The gene is completely included in the deletion, and an SNV is present on the other allele. (**B**) The gene is partially included in the deletion, and an SNV is present on the other allele. (**C**) Presence of an intragenic deletion and an SNV on the other allele.

The most likely consequence of a loss affecting a genomic region with optimally functional genes on the remaining allele is a change in gene expression, which may lead to phenotypic effects. However, if a functional variant also impacts the gene’s function on the other allele (compound heterozygosity), a pathogenic effect is more likely to occur [111]. Therefore, a key genetic mechanism in ASD may involve the co-occurrence of significant variation on both copies of a gene, such as a deletion on one allele and a functional variant on the other. This phenomenon, known as compound heterozygosity, refers to the presence of two different mutations at a given gene locus. Furthermore, the rate of a slightly different type of compound heterozygosity, i.e., two rare loss-of-function sequence variants co-occurring at the same locus (Figure 3), is found to be significantly increased in ASD compared with controls [112,113].

In a study conducted by Lin et al., it is hypothesized that compound heterozygosity of a deletion and a functional sequence variant in the remaining allele occurs more often in ASD patients than in their parents who transmit the deletions. It is supposed that this mechanism of compound heterozygosity may provide an explanation for the penetrance of hereditary CNVs identified in individuals with ASD, compared to unaffected parents. This study aims to provide empirical evidence for the proposed mechanism of compound heterozygosity as a relevant causal factor in a percentage of ASD cases. Indeed, 550 genes were sequenced in 149 individuals with ASD and their parents who transmitted the deletion. Using this method, they identified additional sequence variations present in the remaining allele of the deleted region. It was noted that the co-occurrence of single-nucleotide deletion variants was observed in 13.4% of the probands, compared to 8.1% of the parents. This finding confirms that the presence of sequence variants in the probands was higher than in the parents who transmitted the deletion. The study provides preliminary evidence that some form of compound heterozygosity may play a role in the genetic profile of ASD [114]. This aspect supported the “compound heterozygosity” theory in ASD. Sometimes, the existence of another genetic mutation on the remaining allele may be necessary for some CNVs, especially deletions, to have the potential for causing disease. Thus, a functional variation can be said to “unveil the pathogenicity of a deletion”, indicating a reciprocal relationship implicit in compound nature [115]. According to another study conducted by Bacchelli et al., a proband with ASD was found to have a rare compound heterozygous deletion affecting the *CTNNA3* gene, which codes for αT-catenin. This gene plays an essential role in cell adhesion, which is a major pathway related to ASD and, therefore, is a gene strongly related to the development of this disorder. Segregation analysis and further clinical evaluation in the family have shown that the proband’s unaffected sister is heterozygous for the deletion, having inherited only the paternal deletion. This further supports the impact that a second variant may have in phenotypically determining ASD [116]. Another case of compound heterozygosity was described in a study conducted by Malerba et al.; they report a novel case of a 2.5-year-old female with ASD who is a compound heterozygote for a paternally inherited frameshift and a maternally inherited missense variant in the *GNB5* gene. This study describes the second instance of a patient who is a compound heterozygote for both a missense and a null variation in *GNB5*, confirming the presence of a hotspot for *GNB5* variations in exon 2 (55% of reported pathogenic variants). Homozygous and compound heterozygous pathogenic variants in the *GNB5* gene have recently been associated with a spectrum of clinical presentations ranging from a severe multisystem form of the disorder to a milder form, supporting the idea that the severity of a phenotype is directly related to the type and pathogenic mechanism of the GNB5 gene mutation. In this case, the presence of a compound heterozygous genotype may reduce the clinical manifestations of ASD, rather than exacerbate them [117]. Putatively pathogenic defects found in certain individuals in clinical settings are often inherited from parents who appear unaffected. These findings also support the concept of recessive inheritance in ASD, suggesting that recessive mutations account for 3% of the total risk of ASD. The high heritability of ASD and the fact that most parents of individuals with ASD appear unaffected are consistent with the proposed role of recessive mutations in the disorder [27,118]. In conclusion, compound heterozygosity is one of several mechanisms that explain the variable penetrance of CNVs with known pathogenicity for ASD, resulting in the inconsistent phenotypic expression sometimes observed among carriers of the same suspected pathogenic alteration [114].

Table 5 summarizes the previously described studies that correlate the compound heterozygosity pattern with ASD.

## 4. Conclusions

Biotechnological progress in diagnostic approaches, especially a-CGH and NGS methods, has allowed a great advance in understanding the genetic basis of ASD. As discussed above, ASD is a highly heritable neurodevelopmental disorder, although it is considered a multifactorial condition. Therefore, the genetic component may be one of the main causative factors of ASD and takes into account both common and rare genetic variants as predisposing to the risk of pathogenesis. However, ASD exhibits significant clinical and genetic heterogeneity. Given the etiological complexity and the increasing prevalence of new cases, it is crucial to deepen our understanding of the genetic models associated with this disorder. From this perspective, it becomes possible to identify additional risk factors for ASD, which might typically be linked to specific syndromic conditions. Therefore, accurate and precise genetic diagnosis, extended to family members, is essential for identifying lesser-known genomic variants, especially considering their often variable expressivity and phenotypic penetrance.

Clinical research must be integrated with genetic knowledge of ASD, as a correct clinical classification of ASD patients could allow for their genetic and phenotypic stratification. Therefore, further targeted research, rational use of available case studies, and diagnostic resources will be necessary in order to identify, more accurately, the biological bases of ASD to achieve targeted therapeutic approaches.

## Figures and Tables

**Table 1:** Recurrent CNVs identified in ASD patients.

CNV Locus	Clinical Features Associated with CNV
1q21.1 deletion	Mild-moderate ID, SCZ, mild facial dysmorphic features, microcephaly, cataracts
1q21.1 duplication	Mild-moderate ID, ADHD, mild dysmorphic features, macrocephaly, hypotonia
3q29 deletion	Mild-moderate ID, SCZ, mild facial dysmorphic features
5q35 deletion (Sotos Syndrome)	Developmental delay, microcephaly
7q11.23 duplication (William Syndrome)	ID, SCZ, brain MRI abnormalities, variable dysmorphic features
15q11q13 duplication (Prader-Willi Syndrome)	Mild-moderate ID, epilepsy, ataxia, behavioral problems, hypotonia
15q13.3 deletion	Mild-moderate ID, epilepsy, learning disabilities, ADHD, variable dysmorphic features
16p11.2 deletion	Mild-moderate ID, epilepsy, MCA, variable dysmorphic features, macrocephaly, obesity
16p11.2 duplication	Mild-moderate ID, ADHD, microcephaly, dysmorphic features
16p12.1 deletion	Mild-moderate ID, ADHD, and dysmorphic features
16p13.1 deletion	ID, SCZ, epilepsy, MCA, dysmorphic features
17p11.2 deletion	ID, speech delay, deafness, sleep disturbance, hypotonia
17q12 deletion	Mild-moderate ID, SCZ, epilepsy, facial dysmorphic features
22q11.2 deletion (DiGeorge Syndrome)	ID, SCZ, learning difficulties, MCA, dysmorphic features
22q11.2 duplication	ID, SCZ, speech difficulties, learning difficulties, dysmorphic features, microcephaly

ADHD, attention deficit hyperactivity disorder—ID, intellectual disability—MCA, multiple congenital anomalies—SCZ, schizophrenia—CNS, central nervous system.

**Table 2:** Genetic evidence of the double hit model.

Genetic Alteration	Clinical Effect	References
16p11.2 deletion	Identification of secondary CNVs affecting genes associated with the risk of ASD and intellectual disability, such as *CACNA2D3*, *TRIO*, and *KATNAL2*. Identification of additional rare SNVs that correlate with increased disease severity, especially in multiplex families.	Girirajan et al. [81] Duyzend et al. [82,83] Pizzo et al. [83]
16p12.1 deletion	Secondary genetic events transform the deletion phenotype from normal to severe: - additive double-hit model that explains how the two genomic alterations affect independent pathways with a cumulative effect; - epistatic double-hit model that explains how the two genomic alterations have an impact on the same pathway that is amplified.	Veltman et al. [84] Servetti et al. [89]

**Table 3:** The table shows some genes that act as modifier genes, suggesting that the presence of common variants in multiple genes represents an increased risk of developing NDDs.

Category	Gene	Biological Function	Associated Syndrome/ OMIM Number
Synaptic receptors and cell adhesion molecules	*NRXN1*	Cell-surface receptors that bind neuroligins to form Ca(2+)-dependent neurexin/neuroligin complexes at synapses in the central nervous system.	Pitt-hopkins-like syndrome 2 (#614325)
	*NLGN1*	Neuronal cell surface proteins that may act as splice site-specific ligands for β-neurexins and may be involved in the formation and remodeling of central nervous system synapses.	Susceptibility to autism (#618830)
Scaffolding proteins and the actin cytoskeleton	*SHANK2*	Scaffold protein that localizes to postsynaptic sites of excitatory synapses in the brain.	Susceptibility to autism (#613436)
	*SHANK3*	Scaffolding protein that is enriched in postsynaptic densities of excitatory synapses.	Schizophrenia (#613950) and Phelan-McDermid syndrome (#606232)
Chromatin remodeling and transcription	*MECP2*	Chromatin-associated protein that can both activate and repress transcription. It is required for the maturation of neurons and is developmentally regulated.	Intellectual developmental disorder X-linked syndromic (#300055), Rett syndrome (#312750) and Susceptibility to autism (#300496)
	*SETD5*	Methyltransferase that targets histone H3 lys36 for trimethylation (H3K36me3) and thereby controls the transcriptional landscape in neural progenitors and their derivatives.	Intellectual developmental disorder (#615761)
	*CHD8*	ATP-dependent chromatin-remodeling factor that regulates transcription of β-catenin (*CTNNB1*) target genes.	Intellectual developmental disorder with autism and macrocephaly (#615032)
	*ASH1L*	Histone methyltransferase is involved in epigenetic modification of chromatin and plays important roles in development.	Intellectual developmental disorder (#617796)

**Table 4:** Key points of the incomplete penetrance model of the discussed studies.

Reference	Evidence
de Masfrand et al. [102]	12 patients with NDD presented loss-of-function variants in 11 genes, including *CAMTA1*, *NDUFB11*, and *ZMIZ1*; all variants were inherited from healthy parents, showing phenotypic variability in the same family.
Kendall et al. [108]	Carriers may show intermediate cognitive profiles compared to non-carriers and affected patients. CNV carriers often have lower educational levels and less skilled jobs, despite appearing clinically unaffected.
Furukawa et al. [109]	15q13.3 deletion in affected siblings and a healthy mother who shared the same *CHRNA7* gene deletion, and represents a clear example of incomplete penetrance
Mollon et al. [103]	Additional rare variants influence patient phenotype, and incomplete penetrance interferes with phenotype variability in individuals with CNVs such as 1q21.1, 7q11.23, 16p12.1, 16p11.2, and 22q11.2.
Goh et al. [110]	Penetrance estimates for 92 CNVs using gnomAD v4.0: – High penetrance CNVs duplication of 22q11.2 (51% penetrance), 15q24, 17q11.2, 17q21.31. – Low penetrance or benign CNVs duplication of 15q11.2, proximal duplication of 2q13, deletion of 2q11.2. – “Fully penetrant” CNVs deletion of 7q11.23 < 100% (Williams-Beuren), deletion of 22q11.2 (VCFS).

**Table 5:** Main concepts on compound heterozygosity and its role in ASD.

Study Details	Reference
550 genes sequenced in 149 ASD probands and their deletion-transmitting parents: 13.4% of the probands and 8.1% of the parents had co-occurring variations. The pathogenic effect of a deletion may be triggered by compound heterozygosity.	Lin et al. [114]
The proband with ASD has a compound heterozygous deletion in the *CTNNA3* gene, which is implicated in cell adhesion and is essential in ASD, and his sister acquired only one deletion and is unaffected.	Bacchelli et al. [116]
A 2.5-year-old girl with ASD had compound heterozygous mutations in the *GNB5* gene: a frameshift (paternal) and a missense (maternal) variant. In contrast to homozygous mutations, which result in more severe consequences, compound heterozygosity of *GNB5* might induce milder symptoms of ASD. The severity of mutations in the *GNB5* gene varies.	Malerba et al. [117]

## Data Availability

Not applicable.
